# Association between Serum Essential Metal Elements and the Risk of Schizophrenia in China

**DOI:** 10.1038/s41598-020-66496-7

**Published:** 2020-07-03

**Authors:** Jiahui Ma, Lailai Yan, Tongjun Guo, Siyu Yang, Yaqiong Liu, Qing Xie, Dawei Ni, Jingyu Wang

**Affiliations:** 1 Department of Anesthesiology, Peking University First Hospital, Beijing, 100034 China; 2 Central Laboratory of School of Public Health, Peking University, Beijing, 100191 China; 3 Laboratory for Genome Information Analysis, Center for Integrative Medical Science, RIKEN, Yokohama, 2300045 Japan; 4 Vaccine Research Center, School of Public Health, Peking University, Beijing, 100191 China; 5 Peking University Medical and Health Analysis Center, Peking University, Beijing, 100191 China; 6 Department of Occupational and Environmental Health Sciences, School of Public Health, Peking University, Beijing, 100191 China; 7 Tianjin Center for Disease Control and Prevention, Hedong District, Tianjin, 300171 China; 8 Jiangchuan community health service center, Minhang District, Shanghai, 201100 China

**Keywords:** Metals, Diagnostic markers

## Abstract

Numerous essential metal elements (EMEs) are necessary to maintain the proper function of human body. In this case-control study, we investigated the associations of 11 EMEs [Calcium (Ca), potassium (K), magnesium (Mg), sodium (Na), manganese (Mn), selenium (Se), cobalt (Co), Molybdenum (Mo), copper (Cu), zinc (Zn), and iron (Fe)] in serum with the risk of schizophrenia. We recruited first-episode and drug-naïve schizophrenic patients (cases = 99) and age-sex-matched normal subjects (controls = 99) from Tangshan, Hebei Province, China. The 11 EMEs in serum from cases and controls were quantified by inductively coupled plasma atomic emission spectrometry and inductively coupled plasma mass spectrometry. We observed that a higher level of Mn (OR = 2.390; 95%CI: 1.504–3.796) and lower levels of Ca (OR = 0.939; 95%CI: 0.890–0.990), Mg (OR = 0.806; 95%CI: 0.669–0.972), Na (OR = 0.995; 95%CI: 0.993–0.998), and Se (OR = 0.954; 95%CI: 0.937–0.972) were associated with an elevated risk of schizophrenia. Dose–response relationships between serum EME concentrations and the risk of schizophrenia were observed in most of the schizophrenia-associated EMEs. Moreover, the serum concentrations of these schizophrenia-associated EMEs in patients were correlated with the severity of their clinical symptoms. Significant correlations were found between EMEs and biomarkers associated with schizophrenia related to metabolic and oxidative stress. This study suggested that the concentration and profile of EMEs were different between schizophrenic patients and normal controls and revealed potential metabolisms associated with EMEs and schizophrenia, suggesting EMEs might act as biomarkers of schizophrenia to improve the current situation of diagnosis and treatment.

## Introduction

Schizophrenia is a severe mental disorder associated with multiple risk factors including genetic susceptibility and environmental factors^1^. It occurs in childhood, adolescence or adulthood and leads to disabling conditions which affect the life quality of the patients^2,3^. Although numerous efforts have been made to understand its physiopathology^4–8^, the causes of this disease remain unclear. Further research is required to provide objective diagnosis and effective treatment. Studies have shown that the imbalance of metal elements led to biological malfunctions and nervous system damage, which may be associated with the pathophysiology of schizophrenia^9–11^.

Essential metal elements (EMEs) play essential roles and effects on neurological development through various immunological and metabolic processes^12,13^. A number of studies have shown that the lack of beneficial EMEs and the excess of toxic EMEs are associated with the risk of schizophrenia. For example, reduced Se may cause oxidative stress which is associated with the pathogenesis of schizophrenia^14^. Magnesium has been reported to stimulate and participate in about 300 enzymatic reactions in the body, deficiency of it may be associated with many symptoms of schizophrenia such as apathy, irritability, anxiety and personality changes^15,16^. Indeed, EME supplements such as Se and Mg have been suggested to include in the treatment of schizophrenia^17,18^. The observed difference in the EME concentrations in schizophrenic patients not only provide insight for biomarkers in diagnosis, but to predict the effects of treatment^11,17^. However, the results of the associations between certain EMEs and schizophrenia are inconsistent, while anti-psychotic drugs have been shown to act as a vital confounding factor for the variation of EMEs. For instance, a study on the concentration of 5 elements (Fe, Se, Pb, Cd, and Cr) showed a significantly different distribution between schizophrenic patients with and without anti-psychotic medication^19^. In addition, although several studies have investigated the association between schizophrenia and EMEs, and a large number of studies have explored the pathogenic mechanism of schizophrenia, further research about the role of EMEs in the disease and its association with pathogenic mechanisms is still needed. Therefore, large-scale and more comprehensive studies performed in the first-episode and drug-naïve patients is needed to investigate the association between EMEs and schizophrenia. Therefore, large-scale and more comprehensive studies performed in the first-episode and drug-naïve patients is needed to investigate the association between EMEs and schizophrenia.

In this study, we recruited 99 first-episode and drug-naïve patients with schizophrenia and 99 healthy controls to explore the association between serum concentrations of 11 EMEs (i.e. Ca, K, Mg, Na, Mn, Se, Co, Mo, Zn, Cu, and Fe) and the risk of schizophrenia. The correlations of the 11 EMEs against multiple blood tests as well as oxidative stress biomarkers were also tested to explore the potential metabolisms associated with EMEs and schizophrenia. The findings could help to determine the EMEs variations in Han Chinese schizophrenic patients, and to identify specific EMEs for potential biomarkers of schizophrenia to improve diagnosis and treatment.

## Material and Methods

### Study population and sample preparation

In this study, 99 schizophrenic patients (cases) and 99 healthy subjects (controls) without any known mental disorders were recruited. Patient blood samples were collected from May 2018 to May 2019 in the Tangshan Psychiatric Hospital, Hebei Province, China. All patients included in the study met schizophrenia diagnostic criteria according to the International Classification of Diseases 10^th^ Revision (no. F20). The inclusion criteria were as follows: 18‒60 years of age, live in urban district (mainly in Lunan, Lubei, Guye, and Kaiping district), first-episode and drug-naïve, no history of occupational exposure in heavy industry, no acute infectious diseases, no another psychiatric disorder besides schizophrenia. Age and sex matched control subjects were recruited during the same time in Tangshan Workers’ Hospital. All subjects were confirmed by internal medicine specialist not receiving mineral or vitamin supplements that might have influenced metal levels.

The questionnaire information was collected by trained local health workers as previously described^20^. Collected information included: (1) age; (2) sex; (3) height; (4) weight; (5) single (“no”, “yes”); (6) migration experience (“no”, “yes”); (7) sleep quality (“bad”, “normal”, “good”, “perfect”); (8) birth weight (“2500–2999 g”, “3000–4000 g”, “>4000 g”); (9) family history of schizophrenia (“no”, “yes”); (10) active smoking (“no”, “yes”); (11) drinking (“no”, “yes”); and (12) childhood psychological trauma (“no”, “yes”). Results from blood biochemical and general tests, reflecting glucose metabolism (i.e., fasting blood glucose (FBG)), lipid metabolism (i.e., triglycerides (TG) and total cholesterol (TC)), liver function (i.e., aspartate transaminase (AST), alanine transaminase (ALT), albumin (ALB) and total protein (TP)), renal function (i.e., blood urea nitrogen (BUN), creatinine (CREA) and uric acid (UA)), and blood cell count (i.e. red blood cells (RBC), white blood cells (WBC), platelets (PLT), and hemoglobin (HGB)), were performed and provided by the hospital.

Blood sample (∼3 mL) was collected from subjects after an overnight fasting and stored in a standard blood collection tube (BD Vacutainer® SST™ II Advance). All blood samples were kept at room temperature for about 30 min, and then centrifuged at 3000 rpm for 15 min. Serum samples were then transferred to microcentrifuge tubes (Axygen) and stored at −20 °C until analysis. At the same time, 20 patients were randomly selected to collect 3 mL of fasting venous blood using an EDTA anticoagulant tube. Nucleic acid extraction solution was added at a ratio of 1: 3 (blood: nucleic acid extraction solution), and then stored in a refrigerator at −80 °C protected from light until analysis.

The study protocol was reviewed and approved by the Ethics Review Committee of the Health Science Center, Peking University (IRB00001052–18028). All the participants were aware of the research purpose and signed the informed consent. All methods were performed in accordance with the relevant guidelines and regulations.

### Serum EMEs and whole blood RNA-Sequencing analysis

For determining the concentration of Ca, K, Mg, and Na, each serum sample (0.05 mL) was transferred to a quartz tube, mixed with 4.85 mL 1% freshly prepared ultrapure nitric acid (Merck) and yttrium (Yttrium Standard for ICP-MS, Merck) to a final concentration of 4 ng/mL as an internal standard. Inductively coupled plasma atomic emission spectrometry (ICP-AES, iCAP-6300, Thermo, USA) was used to measure the above 4 EMEs. The wavelengths of ICP-AES selected for Ca, K, Mg and Na were respectively 396.8 nm, 766.4 nm, 279.5 nm and 589.5 nm. For determining the other seven EMEs (Mn, Se, Co, Mo, Cu, Zn, Fe), sample was prepared by mixing 0.1 mL serum sample, 0.1 mL rhodium (20 ng/mL, Merck) and 0.1 mL indium (2 ng/mL), and 1.7 mL 1% nitric acid. To avoid interference, the concentrations of Mn and Se were measured by inductively coupled plasma mass spectrometry (ICP-MS, 7700×, Agilent, USA), while the concentrations of Co, Mo, Cu, Zn, and Fe were measured by another ICP-MS (ELAN DRCII, PerkinElmer, USA). The quality controls applied to this experiment were described previously^20^. The measured and standard concentrations of these certified reference materials were showed in Supplementary Table 1.

RNA quantification and qualification were performed following a standard protocol^21^ which has been described previously^22–24^. Briefly, quality of all the RNA samples have been tested before library construction. RNA degradation, contamination, purity, concentration, and integrity have been assayed as previously described^24^. For samples that meet the RNA quality requirements, 3 µg RNA per sample was used as input material for library construction using the NEBNext® UltraTM RNA Library Prep Kit. After adding an index code, RNA molecules were purified with poly-T oligo-attached magnetic beads. Fragmentation was carried out in the NEBNext First Strand Synthesis Reaction Buffer(5×) supplemented with divalent cations. Then, the first and second strand cDNA were synthesized and the remaining overhangs were converted into blunt ends. The 3’ ends of cDNA fragments were subsequently adenylated and ligated with the NEBNext Adaptor for hybridization. AMPure XP system (Beckman Coulter, Beverly, USA) was used for selecting fragment size of 150~200 bp in length. Selected cDNA fragments were added adaptor and put in 3 µl USER Enzyme (NEB, USA) at 37 °C for 15 min followed by 5 min at 95 °C. Then, PCR products were purified by the AMPure XP system. Library quality was assessed by the Agilent Bioanalyzer 2100 system. After cluster generation with TruSeq PE Cluster Kit v3-cBot-HS (Illumina), 125 bp/150 bp paired-end reads were generated by sequencing library preparations on an Illumina Hiseq platform.

### Statistical analysis

Data was described as median (inter-quartile range). Chi-Squared (χ2) test was used for analyzing the difference of categorical variable and Mann-Whitney U test was used to compare difference of continuity variable. For the EME concentrations comparison among more than two groups, Kruskal-Wallis test was applied. The probability (p) values less than 0.05 were considered significant. The risk of schizophrenia associated with serum EME concentrations was estimated by unconditional logistic regression model. Variables significantly different between case and control were adjusted. Odds ratios (ORs) and 95% confidence intervals (95% CIs) were calculated where a two-tailed *p* value less than 0.05 were considered significant. Spearman correlation was used to calculate the correlation coefficients. Statistical analyses for demographic characteristics and EME concentrations were performed using SPSS ver. 21.0 (SPSS Inc., Chicago, IL, USA).

For sequencing data, clean data were obtained by removing reads containing adapter, reads containing ploy-N and low-quality reads from raw data (FASTQ). At the same time, Q20, Q30 and GC content the clean data were calculated. Above step processed through Perl scrip. All the downstream analyses were based on the clean data with high quality. Reference genome and gene model annotation files were downloaded from genome website directly. Index of the reference genome was built using STAR and paired-end clean reads were aligned to the reference genome using STAR (v2.5.1b). HTSeq v0.6.0 was used to count the reads numbers mapped to each gene. And then CPM of each gene was calculated by reads count mapped to this gene and dividing by the total count of this gene in all samples and then times 1000000. Genes analyzed in this study were based on the literature. Statistical analyses for sequencing data were performed using R 3.6.0 (R Foundation for Statistical Computing, Vienna, Austria, 2019).

## Results

### Population characteristics

In total, 99 schizophrenic patients and 99 healthy subjects were recruited in this study. There is no significant difference between patient with schizophrenia and healthy controls in their ages, sex, BMIs, migration experiences, and smoking and drinking habits. While single status, sleep quality, birth weight, presence of schizophrenia family history and whether experience in psychological trauma in childhood were found as potential risk factors for schizophrenia, which were adjusted in the logistic regression model. Details of the demographic characteristics and distributions between the cases and controls are showed in Table 1.

**Table 1:** Distribution of the characteristics of schizophrenic patients (cases) and healthy subjects (controls).

Characteristics	Cases (*N*^a^ = 99)	Controls (*N* = 99)	*P* ^b^
**Age (years)**			
<25	33 (33.3)	20 (20.2)	0.170
25–30	28 (28.3)	34 (34.3)	
30–35	16 (16.2)	23 (23.2)	
> 35	22 (22.2)	22 (22.2)	
**Sex**			
male	50 (50.5)	43 (43.4)	0.196
female	49 (49.5)	56 (56.6)	
**BMI (kg/m**^**2**^**)**			
<18.5	5 (5.1)	2 (2)	0.700
18.5–23.9	48 (48.5)	50 (50.5)	
24–27.9	33 (33.3)	35 (35.4)	
≥ 28	13 (13.1)	12 (12.1)	
**Single**			
No	48 (48.5)	24 (24.2)	**<0.001**
Yes	51 (51.5)	75 (75.8)	
**Migration experiences**			
No	61 (61.6)	59 (59.6)	0.442
Yes	38 (38.4)	40 (40.4)	
**Sleep quality**			
Bad	20 (20.2)	8 (8.1)	**<0.001**
normal	46 (46.5)	27 (27.3)	
good	24 (24.2)	22 (22.2)	
perfect	9 (9.1)	42 (42.4)	
**Birth weight (g)**^**c**^			
2500–2999	59 (59.6)	33 (33.3)	**0.001**
3000–4000	37 (37.4)	61 (61.6)	
> 4000	3 (3.0)	5 (5.1)	
**Family history (schizophrenia)**			
No	85 (85.9)	96 (97.0)	**0.005**
Yes	14 (14.1)	3 (3.0)	
**Active smoking**			
No	76 (76.8)	72 (72.7)	0.312
Yes	23 (23.2)	27 (27.3)	
**Drinking**			
No	86 (86.9)	92 (92.9)	0.119
Yes	13 (13.1)	7 (7.1)	
Childhood psychological trauma			
No	74 (74.7)	92 (92.9)	**<0.001**
Yes	25 (25.3)	7 (7.1)	

^a^Number of subjects.

^b^Pearson’s chi-square test.

^c^As indicated in the birth certificates.

### Serum EME concentrations

All the 11 EMEs tested in this study showed 100% detection rates from serum. Six EMEs (Ca, K, Mg, Na, Se and Zn) were significantly lower in the case group than the control group (p < 0.05), while two EMEs (Mn and Co) were significant higher in the case group than the control group (Table 2). No significant difference between cases and controls were found for Mo, Cu, and Fe. We further investigated the distribution of EME concentrations in different age and sex group. Significant differences in the concentration of Mg and Mn were observed among different age groups and significant differences in Se and Fe were observed between the male and female groups (Supplementary Table S2 & S3).

**Table 2:** Serum concentrations of EME in schizophrenic patients (cases) and healthy subjects (controls).

EMEs	Cases	Controls	*P* ^a^
Ca^b^	85.18 (82.74–89.4)	88.78 (84.50–93.91)	**<0.001**
K^b^	151.11 (138.08–157.76)	154.45 (144.73–162.60)	**0.032**
Mg^b^	21.95 (20.89–23.27)	22.98 (21.88–24.09)	**0.002**
Na^b^	3148.68 (3096.54–3225.45)	3207.00 (3144.67–3316.33)	**<0.001**
Mn^c^	2.42 (2.03–2.99)	1.97 (1.37–2.43)	**<0.001**
Se^c^	77.98 (68.92–92.53)	111.06 (88.37–126.11)	**<0.001**
Co^c^	1.12 (1.04–1.34)	1.09 (0.96–1.25)	**0.039**
Mo^c^	2.86 (2.53–3.24)	2.87 (2.55–3.27)	0.670
Cu^c^	932.41 (825.64–1007.11)	944.43 (803.84–1089.95)	0.313
Zn^c^	775.22 (697.54–879.01)	840.40 (702.21–951.98)	**0.019**
Fe^c^	1029.82 (819.75–1142.41)	1005.08 (847.70–1265.86)	0.892

^a^In comparison with the median of controls by Mann-Whitney U test.

^b^Serum concentrations of EMEs showed in ug/mL. ^c^Serum concentrations of EMEs showed in ng/mL.

### Associations between EMEs concentrations and schizophrenia risk

**Figure 1:**
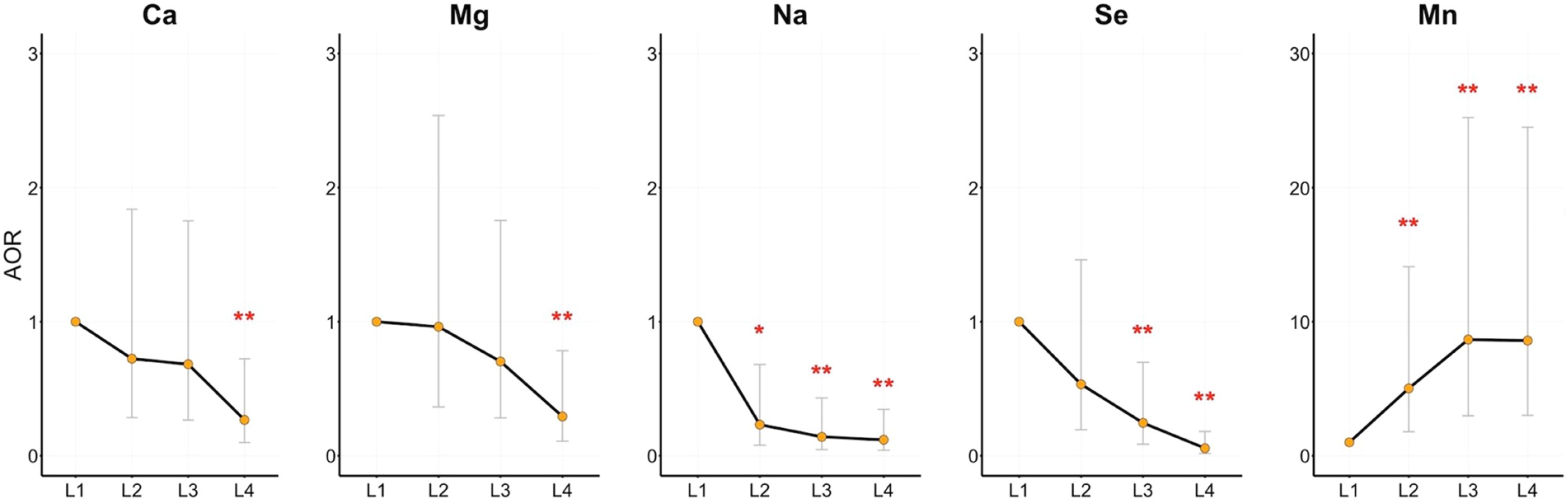
Dose-response relationship between the risk EMEs (Ca, Mg, Na, Se, and Mn) and risk of schizophrenia. Adjusted odds ratios (AORs) for schizophrenia associated with 4 concentration quartiles are represented by the orange dots. Error bar indicated the 95% CI. The four quartiles were calculated from all the 198 subjects according to the concentration of each EME and indicated from low to high as L1, L2, L3, and L4. ^*^p < 0.05 and ^**^p < 0.01.

Odds ratios (ORs) were calculated to quantify the strength of the association between risk of schizophrenia and the concentration of EMEs. The concentrations of Ca, Mg, Na, Mn, and Se were associated with the risk of schizophrenia with and without adjustment by potential confounders, while K and Zn were associated with the risk of schizophrenia crudely (Table 3). The statistically significant adjusted ORs (AORs) of EMEs were: 0.939 [95%CI: 0.890–0.990] for Ca, 0.806 [95%CI: 0.669–0.972] for Mg, 0.995 [95%CI: 0.993–0.998] for Na, 0.954 [95%CI: 0.937–0.972] for Se, and 2.390 [95%CI: 1.504–3.796] for Mn. These results suggested that lower Ca, Mg, Na, and Se concentrations as well as higher Mn concentration were associated with an increased risk of schizophrenia. Concentration of the EMEs were further sub-grouped into quartiles and investigated the dose–response relationship between these EMEs in serum and the risk of schizophrenia. The risk of schizophrenia decreased with increasing levels of Ca, Mg, Na, and Se, while the risk increased with increasing levels of Mn. The detailed dose-response relationships between the schizophrenia risk associated EMEs concentration (Ca, Mg, Na, Mn, and Se) were depicted in Fig. 1 **&** Supplementary Table S4.

**Table 3:** Associations between the prevalence of schizophrenia and the concentrations of EME.

EMEs	Median (IQR)^a^	Univariate OR (95%CI)^d^	*P*^d^	Adjusted OR (95%CI)^e^	*P*^e^
Ca^b^	86.98 (83.46–90.92)	0.923 (0.879–0.968)	**0.001**	0.939 (0.890–0.990)	**0.020**
K^b^	152.75 (142.61–161.11)	0.980 (0.962–0.999)	**0.038**	0.984 (0.962–1.006)	0.153
Mg^b^	22.34 (21.09–23.70)	0.788 (0.673–0.924)	**0.003**	0.806 (0.669–0.972)	**0.024**
Na^b^	3175.24 (3115.72–3268.68)	0.996 (0.994–0.999)	**0.003**	0.995 (0.993–0.998)	**0.003**
Mn^c^	2.19 (1.74–2.72)	2.368 (1.572–3.568)	**<0.001**	2.390 (1.504–3.796)	**<0.001**
Se^c^	91.15 (74.29–114.19)	0.951 (0.936–0.966)	**<0.001**	0.954 (0.937–0.972)	**<0.001**
Co^c^	1.11 (0.99–1.28)	2.163 (0.917–5.104)	0.078	1.903 (0.618–5.854)	0.262
Mo^c^	2.87 (2.53–3.24)	0.974 (0.614–1.547)	0.912	0.659 (0.378–1.150)	0.142
Cu^c^	936.43 (815.05–1060.17)	0.999 (0.997–1.000)	0.144	0.999 (0.997–1.001)	0.172
Zn^c^	807.55 (701.93–916.79)	0.998 (0.996–1.000)	**0.021**	0.999 (0.996–1.001)	0.176
Fe^c^	1008.85 (839.38–1210.66)	1.000 (0.999–1.001)	0.931	1 (0.999–1.001)	0.940

^a^IQR, inter-quartile range.

^b^Unit: ug/mL.

^c^Unit: ng/mL.

^d^Calculated by an unconditional Logistic regression model.

^e^Adjusted OR and 95%CI were calculated by an unconditional Logistic regression model adjusting for the potential confounders, including marital status, sleep quality, birth weight, family history and health-related behavior.

### Correlation between EME concentrations and schizophrenia PANSS scores

**Figure 2:**
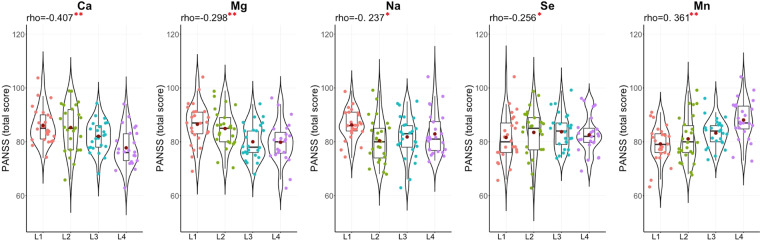
Distributions and correlations of PANSS total score in 4 different quartiles of EME concentration in schizophrenic patients. The four groups were divided by the quartiles of each EME concentration of the 99 patients and recorded as L1, L2, L3, and L4. The red dots indicated the mean total PANSS score in each concentration. Spearman correlation between the total PANSS score and the EME concentration was determined without grouping. The r values were shown at the top. ^*^p < 0.05 and ^**^p < 0.01.

**Figure 3:**
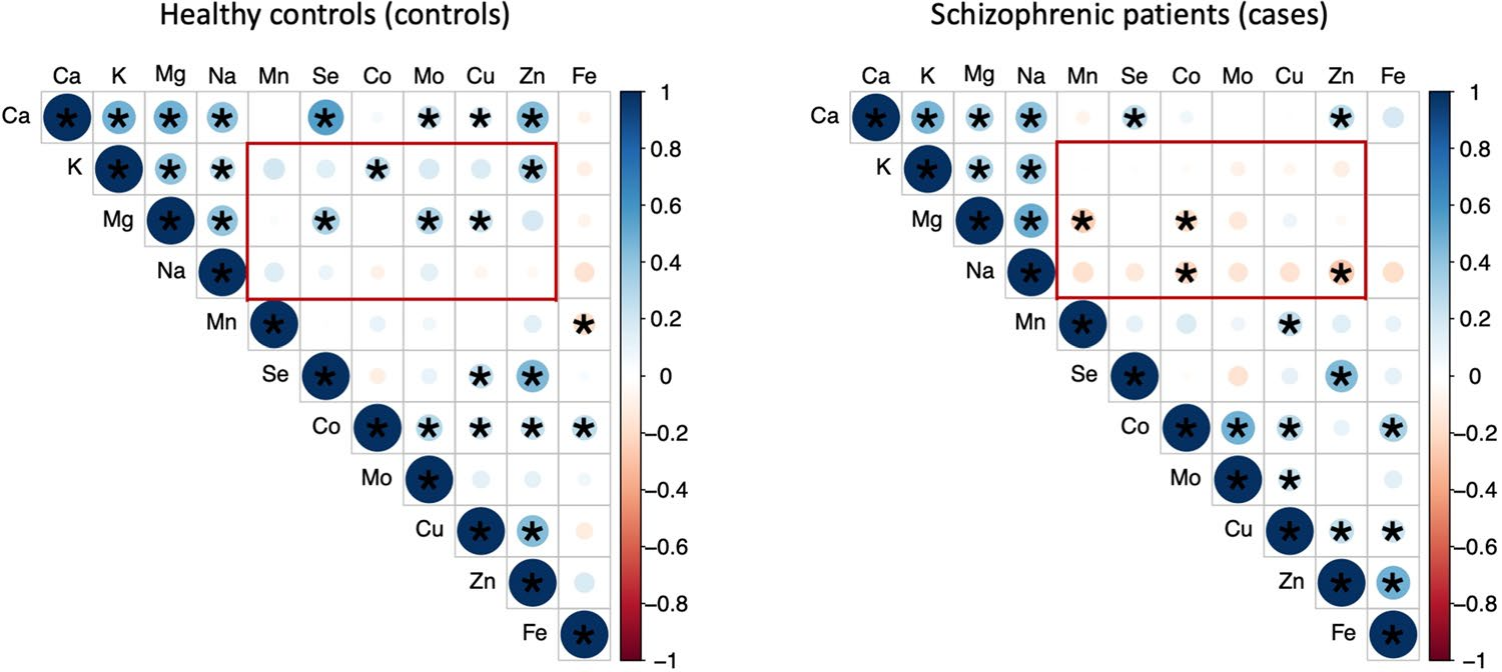
Spearman correlations between EME in cases and controls. Dot with asterisk indicated significant correlation (*p* < 0.05). Dot size and color represented the level of correlation (bigger and darker means greater r). Blue represented positive correlation while red represented negative correlation.

To test whether EME concentration interferes with schizophrenia severity, the Positive and Negative Syndrome Scale (PANSS) was adopted to represent schizophrenia severity^25^. A significant positive correlation was found between the concentration of Mn and the PANSS scores (total and negative scores) while a significant negative correlation was observed between concentration of Ca and the PANSS scores (total and general scores). Besides, concentrations of four EMEs (Mg, Na, Se, and Zn) were negatively correlated with the PANSS total score. (Fig. 2 & Supplementary Table S5**)**. In addition, inter-correlation among the 11 EMEs was also compared between the case and the control groups. More positive and stronger correlations were founded in control group than case group (Fig. 3 & Supplementary Table S6).

### Correlation between EME concentrations and metabolic and oxidative stress biomarkers

**Figure 4:**
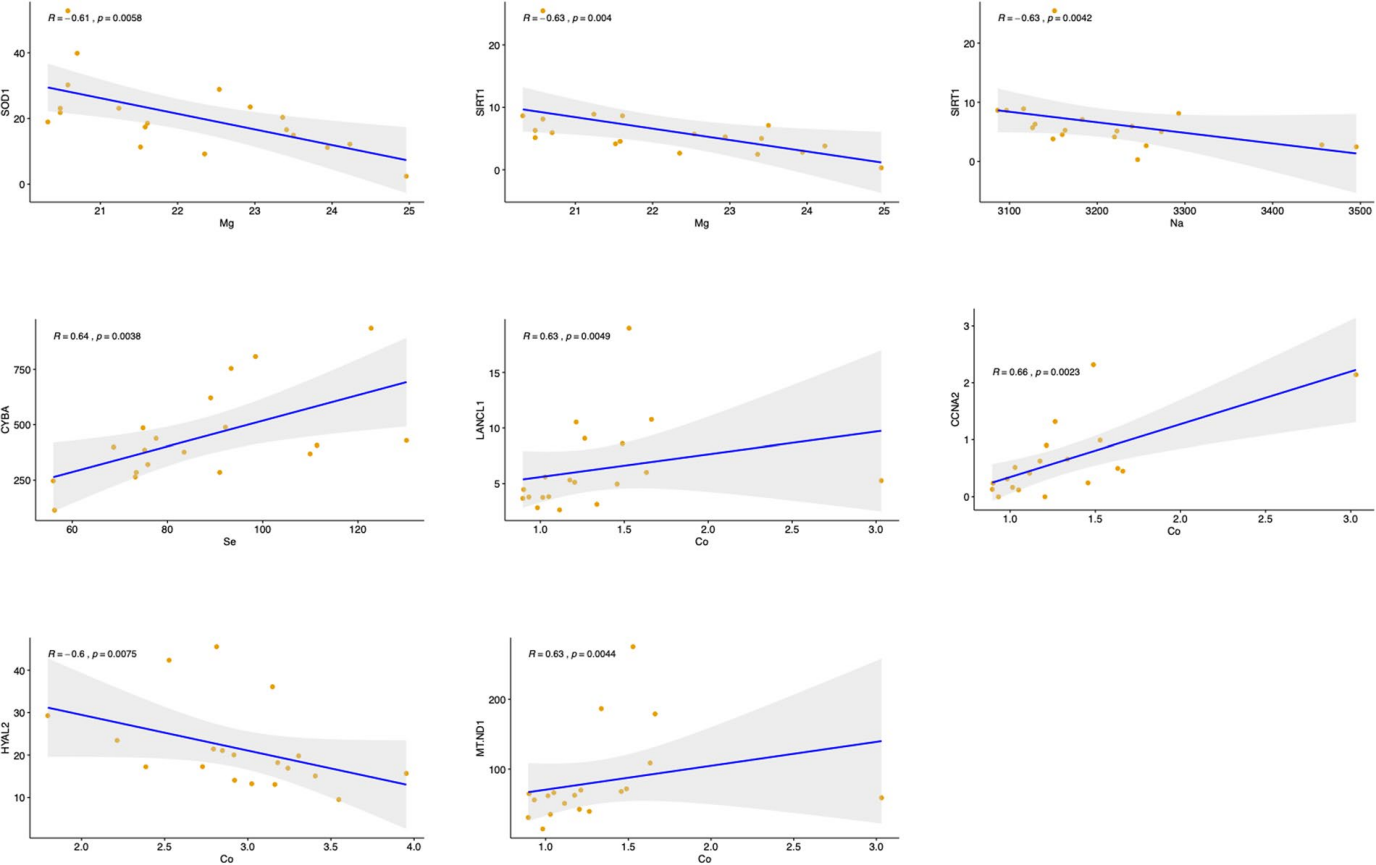
Spearman correlations between EMEs and oxidative stress- and schizophrenia- related genes (r > 0.6). The blue line indicated regression line and 95% confidence interval showed in gray.

Fourteen metabolic biomarkers were selected to suggest biochemistry profile of patients of schizophrenia and healthy controls. Among these 14 biomarkers, level of FBG, TC, ALB, TP, BUN, RBC, and PLT were significantly lower in the patient group than the control group (p < 0.05), while level of ALT was significant higher in the patient group than the control group (Supplementary Table S7). Seventy-three oxidative stress- and schizophrenia- related genes were chosen according to literature to indicate the oxidative stress states in schizophrenic patients. The selection process and result of study inclusion were showed in Supplementary Figure S1. Gene expression level of the schizophrenia patients were determined by RNA-Sequencing where one of the patients was excluded as outlier. Spearman correlation was applied to EME concentrations and above biomarkers (metabolic biomarkers form blood test and oxidative stress biomarker from RNA-sequencing). The results indicated that 7 EMEs were significantly correlated with metabolic biomarkers (Table 4) and all EMEs except Fe have significant correlations with partial oxidative stress markers (Supplementary Table S8). Figure 4 showed the correlation between gene expression level and metal concentration, where only combinations with r value over 0.6 were shown. The concentration of Se was significantly positive correlated with 17 oxidative stress- and schizophrenia- related genes. The gene AKT1 significantly negative correlated to Mn was in the negative regulation of oxidative stress-induced intrinsic apoptotic signaling pathway.

**Table 4:** Spearman correlations (r) between metabolic biomarker/ complete blood count and EMEs.

Parameters	Ca	K	Mg	Na	Mn	Se	Co	Mo	Cu	Zn	Fe
**Glucose metabolism**											
FBG	0.038	−0.055	0.067	−0.047	−0.097	0.250^**^	−0.158	−0.062	0.127	0.167^*^	0.090
**Lipid metabolism**											
TG	−0.126	−0.121	−0.104	−0.178	0.045	−0.024	0.038	0.051	0.171^*^	0.102	0.088
TC	0.125	0.162^*^	0.058	−0.119	−0.096	0.385^**^	−0.107	0.084	0.128	0.229^**^	0.024
**Liver function**											
AST	−0.096	−0.108	−0.105	−0.200	0.012	0.008	−0.122	−0.021	0.091	0.056	0.009
ALT	−0.079	−0.109	−0.082	−0.141	0.041	−0.018	−0.018	0.016	0.135	0.105	0.030
ALB	0.323^**^	0.100	0.151^*^	0.078	−0.041	0.381^**^	−0.003	0.013	0.080	0.290^**^	0.197^**^
TP	0.232^**^	0.055	0.029	−0.084	0.009	0.350^**^	0.091	0.031	0.236^**^	0.224^**^	0.059
**Renal function**											
BUN	0.075	0.066	0.136	0.071	−0.261	0.258^**^	−0.133	−0.097	0.137	0.143	−0.018
UA	0.038	0.072	0.117	0.045	−0.027	0.124	−0.135	−0.081	0.107	0.106	0.050
CREA	0.066	0.002	0.107	−0.042	−0.153	−0.013	0.037	0.135	0.152^*^	0.140	0.131
**Complete blood count**											
RBC	0.232^**^	0.092	0.176^*^	0.038	−0.004	0.247^**^	−0.081	−0.056	0.197^**^	0.362^**^	0.139
WBC	0.035	−0.130	0.036	−0.043	0.001	0.079	−0.064	−0.017	0.086	0.100	−0.030
PLT	0.208^**^	0.174^*^	0.055	0.045	0.087	0.204^**^	−0.053	−0.003	0.070	0.152^*^	−0.097
HGB	0.141^*^	0.014	0.067	−0.045	−0.006	0.141^*^	−0.045	−0.017	0.178^*^	0.327^**^	0.356^**^

*P < 0.05. ^**^p < 0.01.

## Discussion

In this study, we identified 5 schizophrenia risk EMEs, where higher concentration of Mn and lower concentration of Ca, Mg, Na, and Se, in serum were separately associated with an elevated risk of schizophrenia. All these risk EMEs were also correlated with the disease severity (PANSS score). In addition, inter-correlation analysis showed more positive and stronger correlations among EMEs in the control group than the patient group. Overall, the results suggested that the concentration and the profile of the 11 EMEs are different between patients with schizophrenia and healthy controls. Although genetic prevalence act as a major factor in the etiology of schizophrenia, identification of environmental factors, such as EMEs, will provide additional means in diagnosis, treatment design and potential prevention.

The results of the present study suggested that 3 of the bulk EMEs (Mg, Ca and Na) were significantly lower in the patient group than the control group. These bulk EMEs play multiple roles in the central nervous system (CNS) and cognitive function. Their imbalance is able to interfere with the homeostasis, which has showed a key impact on the neurodegenerative and neuropsychiatric disorders, such as Parkinson’s disease, Alzheimer’s disease and schizophrenia^26^. Multiple studies have investigated the relationship between Mg and schizophrenia^27^. A significantly lower intraerythrocyte total Mg concentration has been observed in drug-naïve paranoid schizophrenic patients^28^. In this study, we independently showed a significantly lower serum Mg concentration in schizophrenic patients. In addition to schizophrenia, studies have also suggested an association between the deficiency of Mg and psychiatry diseases^29,30^. The above epidemiological findings have also been supported by the studies of biological mechanisms. In particular, it has been reported that Mg reduces glutamate release and interacts with NMDA receptors^28^, enhancing the GABAergic system^31^, acting as a natural inhibitor of excitotoxicity receptors, and thus preventing neuronal death and the development of schizophrenia^27^. Similar to Mg, a significantly lower level of serum Ca in schizophrenic patients has also been suggested^17^. The deficiency of Ca is involved in the etiology hypothesis of schizophrenia. For example, it has been hypothesized that via a calmodulin-dependent machinery, increased Ca level promoted synthesis of dopamine, which regulates various brain functions^32^. A study based on 2,926 children showed that Ca deficiency further enhanced the toxicity of lead (Pb) to cognitive and behavioral development^33^. Moreover, our study indicated that concentration of Ca was significantly and positively correlated with several metabolic and oxidative biomarkers, suggesting that imbalance in Ca concentration may cause metabolic disorders and dysregulation of the oxidative stress genes associated with schizophrenia. As for Na, several studies have shown that patients with schizophrenia developed symptoms of polydipsia which may cause hyponatremia (low blood Na concentration)^34–36^. In addition, a few studies suggested that severe hyponatremia might lead to cognitive impairment and neurologic disturbances^37,38^. However, a study conducted in another city in China has found different results in terms of Ca and Na, that is, the concentration of Ca and Na were not significantly different between the patients with schizophrenia and healthy controls^39^. The reason may be that in above study 80% recruited patients are not first-episode and drug-naïve. It is noted that Ca and Na may apply to treat mental illness^40,41^. Therefore, concentration of Ca and Na may have been affected by drugs. Secondly, diet is an important factor influencing concentrations of Ca and Na^42,43^. The inconsistence may be caused by dietary differences of the recruited subjects. Taken together, a relationship may exist between the these bulk EMEs (i.e. Ca, Mg, and Na) and the risk of schizophrenia, however, more direct evidence of the underlying mechanism regarding the association will be needed.

Our results showed an association between a higher concentration of Mn and schizophrenia. This echoed our previous study with 114 schizophrenic patients and 114 controls from another region in China where a higher concentration of Mn was found as a risk factor for schizophrenia^44^. In addition, several studies suggested the relationship between alterations of blood (serum or plasma) or hair Mn levels and psychiatry disorders^45–48^. Excessive Mn is widely reported to affect dopamine and other neurotransmitters in the brain^49,50^. The change of neurotransmitter mediated by excessive Mn is also found in schizophrenic patients^49,51^. In animal studies, Mn toxicity has been associated with mitochondrial dysfunction and apoptosis^52,53^, which was also observed in schizophrenia^54^. Our result also observed a significant correlation between concentration of Mn and gene AKT1 which in the apoptosis-related pathways and associated with schizophrenia^55^. On the contrary, some studies suggested that concentration of Mn is lower in the schizophrenic patients than in healthy controls^19,56^. Age is a common factor which may influence an individual’s susceptibility to Mn toxicity^57^. Very young animals as well as humans have increased intestinal Mn absorption^49^ and also have increased accumulations of Mn in the CNS^58^. Meanwhile, our study also suggested a significantly higher Mn level in the younger population. Therefore, more studies with a larger sample size and more focused age range regarding the association between Mn levels and the risk of schizophrenia will better clarify this association.

Selenium is an essential mineral that is incorporated into 25 selenoproteins^59^. In this study, the decreased Se concentration in serum is significantly associated with the risk of schizophrenia, which is consistent with an earlier study^17^. Moreover, Se supplementation has been shown to improve appetite and memory of schizophrenic patients^17^. This agrees with our result showing correlation between Se concentration and schizophrenia severity. Mechanistically, there are two potential explanations for the significant decrease of Se in schizophrenic patients. First, studies have shown that at least half of selenoproteins are involved in suppressing the oxidative stress^60–62^, where oxidative stress is a primary mechanism against neuroprotection^59^ and a factor of the schizophrenia pathophysiology^63^. The correlation between Se and oxidative stress markers related to schizophrenia pathology was also observed in this study. Second, Se / selenoproteins is involved in the dopamine pathways, which cover diverse functions of the CNS such as coordination, memory and cognition^59,64^. Coincident to a previous study^65^, a significant difference of the Se concentration between sex was observed in our study. Although it has been suggested that a specific sexual dimorphism existed in biomarkers of Se status^66^, further investigation should be conducted to uncover the possible mechanisms. In the present study, serum concentration of Se is positively correlated with multiple metabolic biomarkers reflecting glucose, lipid, liver, renal and immune functions (i.e., FBG, TC, ALB, TP, BUN, RBC, PLT, and HGB), which are also significantly different between schizophrenic patients and healthy controls. These results suggest that Se may correlate with large-scale metabolic disorders in schizophrenic patients.

Zinc is essential for brain development, axon function and synaptic transmission. It also involves in nucleic acid metabolism and tubulin expression^67^. In our study, the concentration of Zn showed significant difference between cases and controls but not significant after confounders adjustment. Previous studies about the association of Zn with schizophrenia have obtained inconsistent results. A meta-analysis summarized that the concentration of Zn is associated with the risk of schizophrenia based on 10 studies which included 658 schizophrenic patients and 1008 controls^68^. Another study reported that Zn was found to be significantly associated with schizophrenia in the Asian subgroup, but the results are reversed in the European subgroup^14^. Therefore, the demographics of populations is assumed as a factor of the Zn concentration.

Effects of elements (toxic or beneficial) are often observed when their concentrations are higher or lower than normal control^69^, although the dose response curves for EMEs and human diseases are usually non-linear^70^. This agrees with the dose–response relationship performed in our study, where the higher quartile of Mn significantly increased the risk of schizophrenia compared with the lower quartile. Meanwhile, lower quartiles of Ca, Mg, and Na increased the risk of schizophrenia significantly compared with the higher quartiles. Correlations on EME concentrations and schizophrenia severity further illustrated the association of risk EMEs, where the concentration of Ca, Mg, Na, and Se were inversely correlated with the severity of disease and the concentration of Mn was positively correlated to the severity. Besides, to our knowledge to date, this is the first demonstration that inter-correlation among the 11 EMEs between drug naïve schizophrenic patients and healthy controls was changed, which raises a key question as to whether this alteration of EME profile correlation represents an adaptive or non-adaptive change in the pathophysiological condition associated with schizophrenia.

Three limitations of this study are addressed. First, this study is a cross-sectional study, which is not able to explain the causal relationship between EMEs concentrations and schizophrenia. Second, test of EME concentrations in this study was performed in peripheral blood serum, whether these are representative to human brain or blood cell intracellular EME concentrations is not clear. Third, detection of catecholamines in serum and dopamine alterations and analysis of their correlations with EMEs are not available in this study.

## Conclusions

Our study suggested that increased concentration of Mn and decreased concentration of Ca, Mg, Na and Se in serum samples are associated with the risk of schizophrenia. Trends of the dose–response curve and the correlation between EMEs’ concentrations and total score of PANSS further strengthen this finding. Correlations between EMEs and metabolic and oxidative stress genes associated with schizophrenia has initially clarified their possible role in the pathophysiology of schizophrenia. Overall, this study indicates that the EMEs have the potential to act as biomarkers of schizophrenia to improve the current situation of diagnosis and treatment.

## Supplementary information

Supplementary information
