# Supplementary material for: Association between Serum Essential Metal Elements and the Risk of Schizophrenia in China
Source: Sci Rep. 2020 Jul 3;10:10875. doi: 10.1038/s41598-020-66496-7 (PMC7335092; doi:10.1038/s41598-020-66496-7)
Supplement: Supplementary file 1 — Supplementary information [file 41598_2020_66496_MOESM1_ESM.docx]

**Supplementary Materials**

**Association between Serum Essential Metal Elements and the Risk of Schizophrenia in China**

Jiahui Ma ^1,2,3,4,5,6^, Lailai Yan ^2,4,5^, Tongjun Guo ^6^, Siyu Yang ^7^, Yaqiong Liu ^2^, Qing Xie ^2^, Dawei Ni ^8*^, Jingyu Wang ^2,4,5*^

^1^ Department of Anesthesiology, Peking University First Hospital, Beijing, 100034, China

^2^ Central Laboratory of School of Public Health, Peking University, Beijing, 100191, China

^3^ Laboratory for Genome Information Analysis, Center for Integrative Medical Science, RIKEN, Yokohama, 2300045, Japan

^4^ Vaccine Research Center, School of Public Health, Peking University, Beijing, 100191, China

^5^ Peking University Medical and Health Analysis Center, Peking University, Beijing, 100191, China

^6^ Department of Occupational and Environmental Health Sciences, School of Public Health, Peking University, Beijing, 100191, China

^7^ Tianjin Center for Disease Control and Prevention, Hedong District, Tianjin, 300171, China

^8^ Jiangchuan Community Health Service Center, Minhang District, Shanghai, 201100, China

^*^ Corresponding author:

Jingyu Wang, phone: +0086-10-82801107, E-mail: [wjy@bjmu.edu.cn](mailto:wjy@bjmu.edu.cn)

Dawei Ni, phone: +0086-18117545719, E-mail: kikignil@163.com

**Conflict of Interest:** All authors declare they have no actual or potential competing interests.

Number of tables: 8

Number of figure: 1

**Table S1.** Measured and standard concentrations of the certified reference materials.

| EMEs | Measured value ^a^ | Standard value | Reference type | Coincidence (%) |
| --- | --- | --- | --- | --- |
| Ca ^b^ | 1536±11.79 | 1537 (1469-1605) | Hair | 99.93 |
| K ^b^ | 14.69±0.22 | 14.40^*^ | Hair | 102.01 |
| Mg ^b^ | 249±5.29 | 248 (234-262) | Hair | 100.40 |
| Na ^b^ | 436±4.58 | 445 (405-485) | Hair | 97.98 |
| Mn ^c^ | 31.46±0.99 | 32.7±6.5 | Serum | 92.20 |
| Se ^c^ | 109.67±3.06 | 103±20.8 | Serum | 106.47 |
| Co ^c^ | 3.06±0.04 | 3.13±0.63 | Serum | 97.66 |
| Mo ^c^ | 5.40±0.07 | 5.3±1.06 | Serum | 101.89 |
| Cu ^c^ | 1309±16 | 1360±204 | Serum | 96.25 |
| Zn ^c^ | 1748±25 | 1738±261 | Serum | 100.59 |
| Fe ^c^ | 1379±282 | 1538±154 | Serum | 89.68 |

^a^ The measured value is the mean and standard deviation for three repeated measurements.

^b^ unit: ug/mL.

^c^ unit: ng/mL.

^*^ Reference value.

**Table S2.** Serum concentrations of EME in participants of different age groups.

| EMEs | Age<25 (N=53) | 25≤Age<30 (N=62) | 30≤Age<35 (N=39) | Age≥35 (N=44) | *P* ^a^ |
| --- | --- | --- | --- | --- | --- |
| Ca ^b^ | 86.94 (83.77-91.15) | 87.91 (83.58-92.19) | 88.49 (83.95-90.47) | 85.91 (83.35-90.62) | 0.762 |
| K ^b^ | 155.55 (146.31-162.77) | 150.76 (140.80-160.92) | 150.14 (141.10-160.96) | 153.54 (139.47-159.62) | 0.407 |
| Mg ^b^ | 21.74 (20.57-23.12) | 22.66 (21.79-23.59) | 23.06 (21.61-23.96) | 22.41 (21.09-23.86) | **0.040** |
| Na ^b^ | 3161.22 (3091.07-3240.98) | 3178.79 (3130.72-3297.78) | 3207.00 (3137.20-3325.04) | 3150.56 (3109.77-3243.92) | 0.100 |
| Mn ^c^ | 2.49 (1.86-3.23) | 2.15 (1.71-2.50) | 2.10 (1.73-2.85) | 2.18 (1.72-2.43) | **0.049** |
| Se ^c^ | 82.90 (75.54-99.61) | 95.67 (74.76-115.73) | 90.97 (70.03-129.92) | 94.46 (72.73-119.06) | 0.476 |
| Co ^c^ | 1.14 (1.02-1.34) | 1.10 (0.99-1.35) | 1.09 (0.99-1.16) | 1.10 (0.97-1.28) | 0.167 |
| Mo ^c^ | 2.89 (2.52-3.11) | 2.88 (2.54-3.28) | 2.82 (2.55-3.34) | 2.83 (2.50-3.19) | 0.867 |
| Cu ^c^ | 899.03 (791.14-990.19) | 940.24 (843.94-1069.85) | 1003.16 (880.16-1143.01) | 937.95 (830.47-1049.72) | 0.060 |
| Zn ^c^ | 764.47 (707.06-899.85) | 836.12 (706.64-933.67) | 818.22 (676.24-931.66) | 796.26 (712.19-914.275) | 0.575 |
| Fe ^c^ | 1024.23 (800.22-1209.04) | 1044.95 (818.65-1259.45) | 1005.08 (872.00-1125.37) | 1030.46 (875.89-1204.81) | 0.944 |

^a^ P value of Kruskal-Wallis Test.

^b^ unit: ug/mL.

^c^ unit: ng/mL.

**Table S3.** Serum concentrations of EME in participants of different sex groups.

| EMEs | Male (N=93) | Female (N=105) | *P* ^a^ |
| --- | --- | --- | --- |
| Ca ^b^ | 86.85 (83.40-90.05) | 87.54 (83.54-91.87) | 0.232 |
| K ^b^ | 151.44 (142.70-160.08) | 152.96 (142.30-162.32) | 0.568 |
| Mg ^b^ | 22.30 (21.48-23.67) | 22.35 (20.70-23.81) | 0.497 |
| Na ^b^ | 3181.37 (3113.30-3297.32) | 3174.94 (3117.45-3248.75) | 0.360 |
| Mn ^c^ | 2.15 (1.74-2.71) | 2.20 (1.73-2.74) | 0.977 |
| Se ^c^ | 83.27 (70.53-109.77) | 95.92 (77.33-121.53) | **0.010** |
| Co ^c^ | 1.10 (0.98-1.28) | 1.13 (1.00-1.28) | 0.685 |
| Mo ^c^ | 2.86 (2.51-3.27) | 2.87 (2.56-3.19) | 0.796 |
| Cu ^c^ | 941.96 (814.33-1058.94) | 927.44 (818.13-1062.53) | 0.754 |
| Zn ^c^ | 824.50 (705.42-910.34) | 788.04 (701.65-923.61) | 0.894 |
| Fe ^c^ | 1053.10 (885.48-1403.56) | 962.21 (802.76-1128.49) | **0.006** |

^a^ P value of Mann-Whitney U test.

^b^ unit: ug/mL.

^c^ unit: ng/mL.

**Table S4**. Dose-response relationship between the EME in serum and risk of schizophrenia.

| EMEs | Conc. levels ^a^ | Univariate OR  (95% CI) ^b^ | *P* | Adjusted OR  (95% CI) ^c^ | *P* |
| --- | --- | --- | --- | --- | --- |
| Ca | L1 | 1.000 |  | 1.000 |  |
|  | L2 | 1.053 (0.470-2.358) | 0.901 | 0.724 (0.285-1.839) | 0.497 |
|  | L3 | 0.722 (0.327-1.595) | 0.421 | 0.683 (0.266-1.752) | 0.427 |
|  | L4 | 0.241 (0.103-0.563) ^**^ | **0.001** | 0.267 (0.098-0.723) ^**^ | **0.009** |
| K | L1 | 1.000 |  | 1.000 |  |
|  | L2 | 0.640 (0.289-1.419) | 0.272 | 0.740 (0.293-1.866) | 0.523 |
|  | L3 | 0.921 (0.415-2.043) | 0.839 | 1.123 (0.441-2.862) | 0.808 |
|  | L4 | 0.323 (0.142-0.736) ^**^ | **0.007** | 0.378 (0.141-1.109) | 0.054 |
| Mg | L1 | 1.000 |  | 1.000 |  |
|  | L2 | 0.693 (0.311-1.543) | 0.369 | 0.962 (0.364-2.538) | 0.937 |
|  | L3 | 0.664 (0.299-1.472) | 0.313 | 0.703 (0.282-1.755) | 0.451 |
|  | L4 | 0.297 (0.130-0.679) ^**^ | **0.004** | 0.293 (0.109-0.784) ^*^ | **0.015** |
| Na | L1 | 1.000 |  | 1.000 |  |
|  | L2 | 0.397 (0.171-0.925) ^*^ | **0.032** | 0.232 (0.079-0.681) ^**^ | **0.001** |
|  | L3 | 0.215 (0.092-0.505) ^**^ | **<0.001** | 0.141 (0.046-0.431) ^**^ | **0.008** |
|  | L4 | 0.187 (0.079-0.443) ^**^ | **<0.001** | 0.119 (0.041-0.346) ^**^ | **0.001** |
| Mn | L1 | 1.000 |  | 1.000 |  |
|  | L2 | 5.150 (2.065-12.844) ^**^ | **<0.001** | 5.027 (1.792-14.100) ^**^ | **0.002** |
|  | L3 | 7.433 (2.962-18.653) ^**^ | **<0.001** | 8.668 (2.979-25.223) ^**^ | **<0.001** |
|  | L4 | 9.396 (3.683-23.969) ^**^ | **<0.001** | 8.594 (3.014-24.506) ^**^ | **<0.001** |
| Se | L1 | 1.000 |  | 1.000 |  |
|  | L2 | 0.471 (0.190-1.168) | 0.104 | 0.533 (0.194-1.462) | 0.222 |
|  | L3 | 0.167 (0.068-0.408) ^**^ | **<0.001** | 0.245 (0.086-0.697) ^**^ | **0.008** |
|  | L4 | 0.042 (0.014-0.12) ^**^ | **<0.001** | 0.056 (0.017-0.181) ^**^ | **<0.001** |
| Co | L1 | 1.000 |  | 1.000 |  |
|  | L2 | 2.815 (1.244-6.369) ^*^ | **0.013** | 4.437 (1.659-11.870) ^**^ | **0.003** |
|  | L3 | 2.103 (0.939-4.710) | 0.071 | 3.929 (1.429-10.797) ^**^ | **0.008** |
|  | L4 | 2.382 (1.058-5.366) ^*^ | **0.036** | 2.757 (1.056-7.201) ^*^ | **0.038** |
| Mo | L1 | 1.000 |  | 1.000 |  |
|  | L2 | 0.886 (0.403-1.949) | 0.764 | 0.654 (0.254-1.685) | 0.38 |
|  | L3 | 1.084 (0.494-2.377) | 0.841 | 0.873 (0.341-2.232) | 0.777 |
|  | L4 | 0.752 (0.341-1.658) | 0.480 | 0.555 (0.220-1.403) | 0.214 |
| Cu | L1 | 1.000 |  | 1.000 |  |
|  | L2 | 1.702 (0.768-3.774) | 0.190 | 1.362 (0.517-3.588) | 0.532 |
|  | L3 | 1.621 (0.735-3.573) | 0.231 | 1.614 (0.626-4.162) | 0.322 |
|  | L4 | 0.682 (0.305-1.523) | 0.350 | 0.566 (0.212-1.513) | 0.257 |
| Zn | L1 | 1.000 |  | 1.000 |  |
|  | L2 | 2.500 (1.088-5.742) ^*^ | **0.031** | 2.106 (0.795-5.580) | 0.134 |
|  | L3 | 0.923 (0.421-2.022) | 0.841 | 0.960 (0.379-2.433) | 0.932 |
|  | L4 | 0.441 (0.194-1.004) | 0.051 | 0.589 (0.228-1.521) | 0.274 |
| Fe | L1 | 1.000 |  | 1.000 |  |
|  | L2 | 0.752 (0.341-1.658) | 0.480 | 0.786 (0.312-1.977) | 0.609 |
|  | L3 | 1.385 (0.627-3.058) | 0.421 | 2.402 (0.899-6.414) | 0.080 |
|  | L4 | 0.692 (0.313-1.529) | 0.363 | 0.819 (0.315-2.129) | 0.682 |

UOR, univariate OR; AOR, adjusted OR; ^*^ *p* < 0.05 and ^**^ *p* < 0.01.

^a^ The EME concentration was classified into four levels by 25%, 50%, and 75% quartiles, noted as L1, L2, L3 and L4.

^b^ Calculated by using an unconditional Logistic regression model.

^c^ Calculated by using an unconditional Logistic regression model adjusting for a marital status, sleep quality, birth weight, family history and health-related behavior.

**Table S5.** Spearman correlations between EMEs in cases and controls (r).

| EMEs | Ca | K | Mg | Na | Mn | Se | Co | Mo | Cu | Zn | Fe |
| --- | --- | --- | --- | --- | --- | --- | --- | --- | --- | --- | --- |
| Ca | 1 | **0.468^**^** | **0.328^**^** | **0.419^**^** | -0.078 | **0.263^**^** | 0.075 | 0.019 | 0.03 | **0.274^**^** | 0.189 |
| K | **0.481^**^** | 1 | **0.315^**^** | **0.389^**^** | 0.02 | -0.024 | -0.031 | -0.09 | -0.062 | -0.11 | 0.016 |
| Mg | **0.488^**^** | **0.419^**^** | 1 | **0.502^**^** | **-0.252^*^** | 0.01 | **-0.206^*^** | -0.137 | 0.086 | -0.047 | 0.007 |
| Na | **0.413^**^** | **0.276^**^** | **0.404^**^** | 1 | -0.179 | -0.137 | **-0.212^*^** | -0.151 | -0.164 | **-0.270^**^** | -0.18 |
| Mn | 0.018 | 0.195 | 0.039 | 0.159 | 1 | 0.119 | 0.165 | 0.084 | **0.260^**^** | 0.142 | 0.103 |
| Se | **0.557^**^** | 0.147 | **0.325^**^** | 0.092 | -0.023 | 1 | -0.03 | -0.165 | 0.115 | **0.448^**^** | 0.12 |
| Co | 0.057 | **0.261^**^** | -0.001 | -0.081 | 0.103 | -0.105 | 1 | **0.483^**^** | **0.285^**^** | 0.106 | **0.334^**^** |
| Mo | **0.242^*^** | 0.176 | **0.310^**^** | 0.128 | 0.076 | 0.104 | 0.299^**^ | 1 | **0.226^*^** | 0.018 | 0.136 |
| Cu | **0.199^*^** | 0.162 | **0.235^*^** | -0.068 | 0.005 | **0.253^*^** | **0.226^*^** | 0.129 | 1 | **0.259^**^** | **0.230^*^** |
| Zn | **0.438^**^** | **0.336^**^** | 0.182 | -0.052 | 0.131 | **0.450^**^** | **0.243^*^** | 0.115 | 0.432^**^ | 1 | **0.488^**^** |
| Fe | -0.071 | -0.094 | -0.071 | -0.166 | -.203^*^ | 0.047 | **0.266^**^** | 0.073 | -0.119 | 0.171 | 1 |

^*^ P<0.05

^**^ P<0.01

The lower-left corner with grey background represents the correlations of ETMs in controls while the upper-right corner with white background represents the correlations of ETMs in cases.

**Table S6.** Spearman correlations of PANSS score and concentration of EMEs in schizophrenic patients (r).

| EMEs | PANSS score (Positive) | PANSS score (Negative) | PANSS score (General) | PANSS score (Total) |
| --- | --- | --- | --- | --- |
| Ca | -0.082 | -0.133 | **-0.220^*^** | **-0.407^**^** |
| K | 0.075 | 0.056 | -0.079 | -0.004 |
| Mg | -0.058 | -0.127 | -0.197 | **-0.298^**^** |
| Na | 0.026 | -0.044 | -0.19 | **-0.237^*^** |
| Mn | 0.189 | **0.258^**^** | 0.085 | **0.361^**^** |
| Se | -0.092 | -0.092 | -0.125 | **-0.256^*^** |
| Co | 0.148 | -0.082 | 0.144 | 0.146 |
| Mo | 0.09 | 0.058 | -0.128 | -0.022 |
| Cu | 0.043 | 0.032 | 0.024 | 0.031 |
| Zn | -0.165 | -0.123 | 0.003 | **-0.199^*^** |
| Fe | -0.119 | -0.049 | 0.109 | 0.058 |

^*^ P<0.05

^**^ P<0.01

**Table S7.** Distribution of the metabolic biomarker/ complete blood count between cases and controls.

| Metabolic biomarker | Cases | Controls | P ^a^ |
| --- | --- | --- | --- |
| Glucose metabolism | | | |
| FBG (mmol/L); median (IQR) | 4.60 (4.30-5.20) | 5.12 (4.71-5.72) | **<0.001** |
| Lipid metabolism | | | |
| TG (mmol/L); median (IQR) | 1.44 (1.01-2.15) | 1.27 (0.97-1.83) | 0.249 |
| TC (mmol/L); median (IQR) | 4.13 (3.55-4.65) | 4.82 (4.29-5.72) | **<0.001** |
| Liver function | | | |
| AST (U/L); median (IQR) | 17 (15-22) | 18 (15-21) | 0.709 |
| ALT (U/L); median (IQR) | 18 (13-29) | 15 (12-20) | **0.004** |
| ALB (g/L); median (IQR) | 40.00 (38.00-43.00) | 42.12 (39.46-44.89) | **<0.001** |
| TP (g/L); median (IQR) | 68.00 (64.00-71.00) | 69.89 (64.07-76.39) | **0.015** |
| Renal function | | | |
| BUN (mmol/L); median (IQR) | 4.08 (3.34-5.16) | 5.24 (4.42-6.45) | **<0.001** |
| CREA (umol/L); median (IQR) | 65.00 (57.00-72.00) | 64.05 (54.50-73.18) | 0.885 |
| UA (umol/L); median (IQR) | 282.00 (217.00-353.00) | 262.00 (217.50-363.75) | 0.840 |
| Complete blood count |  |  |  |
| RBC (10^12/L); median (IQR) | 4.18 (3.87-4.63) | 4.46 (4.24-4.72) | **<0.001** |
| WBC (10^9/L); median (IQR) | 5.50 (4.77-7.09) | 5.66 (4.96-7.13) | 0.226 |
| PLT (10^9/L); median (IQR) | 227.00 (199.00-272.00) | 252.00 (215.00-295.00) | **<0.001** |
| HGB (g/L); median (IQR) | 133.00 (121.00-142.00) | 133.00 (125.00-142.00) | 0.524 |

^a^ In comparison with the median of controls by Mann-Whitney U test.

| **Table S8.** Spearman correlations (r) between oxidative stress- and schizophrenia- related genes and EMEs. | | | | | | | | | | | | |
| --- | --- | --- | --- | --- | --- | --- | --- | --- | --- | --- | --- | --- |
| Gene | Related pathway ^a^ | Ca | K | Mg | Na | Mn | Se | Co | Mo | Cu | Zn | Fe |
| BAD | RHP | 0.060 | 0.068 | -0.343 | -0.414 | -0.056 | 0.181 | 0.244 | 0.063 | 0.030 | 0.288 | 0.098 |
| PDK2 | CRROS | 0.330 | 0.076 | 0.123 | -0.160 | -0.215 | 0.167 | 0.263 | 0.072 | 0.195 | -0.090 | 0.223 |
| ERCC1 | ROS | **0.525^*^** | -0.019 | -0.295 | 0.151 | -0.130 | 0.386 | 0.326 | -0.116 | -0.156 | 0.191 | 0.382 |
| HGF | NRHPCD | -0.058 | -0.302 | 0.220 | -0.121 | 0.158 | 0.137 | 0.049 | -0.202 | 0.458 | 0.200 | 0.018 |
| ABCC2 | ROS | -0.097 | -0.035 | -0.203 | -0.176 | 0.329 | 0.204 | 0.025 | 0.062 | 0.371 | 0.065 | -0.411 |
| BAK1 | RHP | 0.344 | 0.198 | -0.443 | -0.282 | 0.114 | 0.437 | **0.591^**^** | 0.118 | 0.084 | 0.004 | 0.209 |
| PNKP | ROS | 0.242 | 0.144 | -0.176 | 0.123 | 0.007 | 0.391 | 0.202 | **-0.516^*^** | 0.004 | 0.047 | -0.116 |
| CYBA | CROS | 0.226 | 0.019 | -0.319 | -0.104 | 0.105 | **0.642^**^** | 0.363 | -0.095 | 0.086 | 0.311 | 0.139 |
| PTPRN | RROS | 0.105 | -0.249 | 0.077 | 0.419 | -0.079 | -0.116 | -0.104 | -0.239 | 0.009 | -0.016 | 0.137 |
| NDUFB4 | ROS | 0.239 | 0.353 | -0.348 | -0.246 | -0.032 | 0.204 | 0.358 | -0.033 | 0.158 | 0.005 | -0.009 |
| HYAL2 | RROS | 0.067 | -0.323 | 0.238 | 0.011 | -0.014 | 0.307 | 0.040 | **-0.602^**^** | 0.147 | 0.286 | -0.053 |
| PTGS2 | ROS | 0.033 | 0.146 | -0.389 | **-0.558^*^** | 0.007 | 0.230 | 0.318 | -0.167 | 0.088 | 0.289 | 0.140 |
| EDN1 | RO | -0.242 | 0.012 | 0.172 | -0.344 | -0.133 | 0.024 | 0.007 | -0.133 | -0.197 | 0.153 | -0.155 |
| PTGS1 | ROS | **0.528^*^** | -0.014 | 0.017 | 0.233 | -0.125 | 0.367 | 0.289 | -0.039 | 0.081 | -0.146 | 0.081 |
| SIRT1 | ROS; RHP; CRHP; NROSA | -0.037 | -0.046 | **-0.628^**^** | **-0.626^**^** | 0.105 | 0.381 | 0.406 | 0.182 | -0.034 | 0.376 | 0.168 |
| HNRNPM | RMS | 0.011 | -0.346 | -0.156 | -0.221 | 0.096 | **0.481^*^** | -0.002 | **-0.519^*^** | 0.146 | 0.326 | -0.160 |
| PRODH | IASPROS | 0.085 | 0.176 | 0.084 | 0.312 | -0.212 | -0.044 | 0.014 | -0.312 | -0.282 | -0.447 | -0.094 |
| APEX1 | CRHP | 0.368 | 0.323 | -0.421 | -0.235 | -0.012 | 0.430 | 0.419 | 0.056 | 0.079 | -0.007 | 0.174 |
| CST3 | CRHP | **0.519^*^** | 0.084 | -0.151 | 0.139 | 0.223 | **0.526^*^** | 0.302 | -0.004 | **0.530^*^** | -0.037 | 0.188 |
| MAP1LC3A | CRHP | 0.054 | -0.125 | -0.058 | -0.389 | -0.151 | 0.228 | 0.011 | -0.209 | -0.123 | 0.253 | -0.204 |
| MYEF2 | RMS | -0.096 | -0.456 | 0.014 | -0.242 | 0.339 | 0.381 | -0.002 | -0.389 | 0.323 | 0.453 | -0.065 |
| HSPB1 | NROSA | -0.032 | 0.040 | -0.001 | 0.160 | -0.330 | -0.263 | 0.121 | -0.400 | -0.211 | -0.153 | -0.002 |
| PPIF | CRHP | 0.377 | 0.193 | -0.291 | -0.307 | -0.172 | 0.435 | **0.512^*^** | -0.105 | 0.030 | 0.177 | 0.153 |
| COL1A1 | RHP | -0.127 | -0.173 | 0.014 | -0.044 | -0.125 | 0.038 | 0.141 | **-0.519^*^** | -0.299 | -0.057 | 0.123 |
| PDGFRB | CRHP | 0.054 | -0.039 | -0.232 | 0.304 | 0.116 | 0.077 | 0.198 | 0.005 | 0.065 | -0.107 | 0.174 |
| LANCL1 | ROSND | 0.088 | 0.272 | -0.382 | -0.384 | -0.088 | 0.328 | **0.628^**^** | -0.051 | 0.047 | 0.086 | 0.161 |
| TPO | ROS | -0.016 | -0.174 | 0.049 | 0.026 | 0.040 | 0.138 | -0.333 | -0.009 | -0.076 | 0.290 | 0.026 |
| NFE2L2 | CROS; PRTROS; CRHP; NROSA; NRHPCD; RRSR | 0.111 | -0.202 | -0.195 | -0.423 | -0.065 | 0.332 | 0.360 | 0.077 | 0.067 | 0.289 | 0.163 |
| PKD2 | CRROS | 0.314 | -0.018 | -0.117 | -0.196 | 0.326 | **0.509^*^** | 0.010 | -0.192 | 0.397 | 0.452 | 0.017 |
| DUSP1 | RHP | **0.579^*^** | -0.114 | 0.182 | 0.112 | 0.261 | **0.500^*^** | -0.160 | -0.256 | 0.430 | 0.477^*^ | 0.272 |
| PRDX5 | ROS; CRROS; CROS | 0.146 | 0.184 | 0.189 | -0.070 | -0.396 | -0.274 | 0.125 | -0.028 | -0.386 | -0.054 | 0.179 |
| KLF2 | CRHP | 0.316 | -0.014 | -0.284 | 0.170 | -0.042 | 0.368 | 0.391 | -0.070 | -0.098 | 0.188 | 0.24 |
| APOE | RROS | 0.222 | 0.116 | 0.150 | 0.370 | -0.207 | 0.088 | 0.066 | -0.164 | -0.157 | -0.097 | 0.205 |
| IL6 | CRHP | 0.000 | 0.052 | 0.032 | -0.152 | 0.202 | 0.334 | -0.076 | -0.378 | 0.395 | 0.258 | -0.241 |
| KLF4 | CRHP | 0.249 | -0.249 | -0.254 | -0.105 | -0.218 | 0.360 | 0.275 | -0.261 | -0.068 | 0.237 | 0.254 |
| DUOX1 | ROS | 0.082 | 0.191 | **-0.498^*^** | -0.229 | 0.281 | **0.481^*^** | 0.319 | -0.183 | 0.113 | 0.121 | -0.125 |
| HNRNPD | CRNO | 0.007 | -0.323 | -0.339 | -0.349 | 0.074 | 0.395 | 0.254 | -0.135 | 0.088 | 0.398 | 0.193 |
| SOD1 | RS; SDA; RSR; CROS; RHP; PROSA; | 0.061 | 0.089 | **-0.608^**^** | -0.221 | 0.191 | 0.272 | 0.437 | 0.114 | 0.074 | -0.221 | -0.104 |
| APP | ROS | **0.491^*^** | 0.026 | **-0.537^*^** | 0.109 | 0.419 | **0.574^*^** | 0.323 | 0.202 | 0.204 | 0.002 | 0.053 |
| AKT1 | ROS; CRROS; NROSA | 0.346 | -0.089 | 0.050 | -0.06 | **-0.479^*^** | 0.274 | 0.418 | -0.228 | -0.174 | 0.061 | 0.249 |
| MCL1 | PROSNA | 0.105 | -0.088 | -0.348 | -0.335 | 0.189 | **0.460^*^** | **0.554^*^** | -0.063 | 0.272 | 0.125 | 0.014 |
| RHOB | CRHP | 0.209 | 0.119 | -0.326 | -0.282 | 0.214 | 0.456 | 0.307 | 0.095 | 0.093 | 0.219 | 0.012 |
| HSPD1 | RHP | **0.579^*^** | 0.133 | -0.277 | 0.012 | 0.133 | **0.588^**^** | 0.353 | -0.154 | 0.237 | 0.049 | 0.265 |
| SNCA | CROS | 0.446 | -0.112 | -0.069 | -0.054 | 0.167 | **0.505^*^** | -0.118 | -0.070 | 0.282 | 0.072 | -0.005 |
| CCNA2 | CRNO | -0.079 | 0.047 | -0.153 | -0.304 | 0.290 | -0.018 | **0.656^**^** | 0.111 | **0.489^*^** | -0.042 | 0.300 |
| RPS3 | ROS; CRHP | 0.184 | 0.009 | -0.435 | -0.137 | 0.181 | **0.582^*^** | 0.228 | 0.049 | -0.025 | 0.251 | 0.156 |
| RBPMS | ROS | -0.114 | -0.166 | 0.417 | 0.009 | -0.365 | 0.066 | -0.005 | **-0.480^*^** | 0.007 | 0.017 | -0.132 |
| PINK1 | CROS; RCROS; NROSCD; NROSND; NRHPNA; NRAHP | 0.323 | 0.107 | 0.266 | 0.184 | 0.082 | 0.082 | **-0.493^*^** | -0.119 | 0.277 | -0.033 | -0.186 |
| ATP13A2 | CROS | 0.268 | -0.082 | -0.119 | -0.132 | -0.186 | **0.511^*^** | 0.112 | -0.261 | -0.274 | 0.288 | 0.107 |
| CYBB | CROS | 0.419 | 0.088 | -0.312 | -0.111 | 0.088 | 0.398 | 0.189 | -0.070 | 0.193 | -0.009 | 0.181 |
| GPX4 | ROS | 0.139 | 0.226 | **-0.568^*^** | 0.016 | 0.409 | 0.451 | **0.470^*^** | 0.144 | 0.377 | 0.012 | -0.261 |
| EEF2 | RHP | **0.489^*^** | 0.040 | -0.354 | 0.142 | 0.132 | **0.537^*^** | 0.065 | 0.082 | 0.03 | 0.142 | 0.202 |
| PRDX2 | ROS; RSR; CROS; | -0.061 | -0.137 | 0.408 | 0.116 | -0.168 | -0.165 | -0.089 | 0.019 | 0.353 | -0.021 | 0.081 |
| GPR37L1 | NRHPCD | -0.146 | -0.265 | -0.103 | 0.195 | 0.074 | 0.218 | -0.187 | -0.158 | 0.145 | 0.211 | -0.167 |
| FOS | CRROS | 0.125 | -0.298 | 0.019 | 0.196 | 0.426 | 0.219 | -0.116 | -0.404 | **0.491^*^** | 0.237 | 0.009 |
| CYCS | CROS | -0.065 | -0.125 | -0.268 | -0.209 | 0.225 | 0.246 | 0.228 | -0.277 | 0.147 | -0.021 | 0.03 |
| NET1 | CRHP | 0.126 | -0.172 | -0.408 | -0.058 | -0.063 | 0.374 | 0.258 | -0.046 | -0.253 | -0.016 | 0.063 |
| MSRB3 | ROS | 0.081 | -0.242 | -0.287 | -0.195 | 0.323 | 0.316 | **0.525^*^** | 0.237 | 0.153 | 0.084 | 0.177 |
| FOSL1 | RHP | 0.269 | 0.031 | -0.255 | -0.097 | -0.180 | 0.312 | 0.348 | -0.403 | -0.193 | 0.101 | 0.361 |
| JUN | CRROS; RHP | **0.467^*^** | -0.049 | -0.37 | 0.023 | 0.116 | **0.563^*^** | -0.068 | -0.160 | -0.089 | 0.274 | 0.188 |
| PSAP | NRHPCD | **0.516^*^** | 0.105 | -0.198 | 0.054 | 0.230 | **0.509^*^** | 0.212 | -0.177 | 0.407 | -0.077 | 0.007 |
| MT-ND6 | RHP | -0.107 | -0.333 | -0.301 | -0.132 | 0.346 | 0.163 | 0.177 | 0.098 | 0.128 | -0.004 | 0.107 |
| MT-ND5 | RHP | -0.377 | -0.456 | -0.152 | 0.030 | 0.070 | -0.086 | 0.335 | -0.230 | 0.044 | -0.168 | -0.016 |
| MT-CO1 | ROS | 0.177 | -0.198 | -0.116 | 0.023 | -0.144 | 0.239 | **0.475^*^** | 0.009 | -0.089 | 0.128 | 0.375 |
| MT-ND3 | ROS | -0.24 | -0.105 | -0.273 | -0.109 | 0.330 | -0.056 | **0.563^*^** | -0.021 | 0.360 | -0.281 | 0.065 |
| MT-ND1 | RH | -0.275 | -0.121 | -0.310 | 0.132 | 0.174 | -0.147 | **0.633^*^** | -0.307 | 0.137 | -0.281 | 0.125 |
| HSPA1B | CROS | 0.360 | 0.088 | -0.158 | 0.454 | 0.091 | 0.046 | 0.140 | -0.442 | 0.063 | -0.304 | 0.104 |
| HSPA1A | CROS | 0.232 | -0.023 | -0.239 | -0.172 | 0.135 | **0.635^**^** | 0.237 | -0.316 | 0.054 | 0.347 | 0.025 |
| AIF1 | CROS; CRH | 0.012 | 0.260 | **-0.488^*^** | -0.028 | 0.163 | 0.177 | 0.384 | -0.354 | 0.198 | -0.104 | -0.277 |
| GPX3 | RLP; CROS | 0.256 | 0.044 | -0.404 | -0.432 | 0.281 | **0.540^*^** | 0.198 | -0.025 | -0.046 | 0.453 | 0.154 |
| TREX1 | CRROS | 0.137 | 0.214 | -0.056 | 0.198 | -0.288 | -0.098 | 0.205 | -0.379 | 0.095 | -0.356 | -0.081 |
| GPX1 | RH; CROS; RHP; NROSA | 0.046 | 0.165 | 0.079 | 0.144 | -0.054 | -0.232 | -0.181 | -0.081 | 0.012 | 0.049 | 0.123 |
| HP | RHP | -0.314 | **-0.474^*^** | -0.094 | -0.132 | 0.289 | -0.005 | 0.195 | -0.384 | 0.421 | 0.174 | 0.030 |

^*^ All the related pathways listed are sub-class pathways of response to oxidative stress;

RHP: response to hydrogen peroxide; CRROS: cellular response to reactive oxygen species; ROS: response to oxidative stress; CRHP: cellular response to hydrogen peroxide; NRHPCD: negative regulation of hydrogen peroxide-mediated programmed cell death; CROS: cellular response to oxidative stress; RCROS: regulation of cellular response to oxidative stress; RROS: response to reactive oxygen species; RSR: removal of superoxide radicals; CRNO: cellular response to nitric oxide; RO: response to ozone; RMS: regulation of mRNA stability involved in response to oxidative stress; IASPROS: intrinsic apoptotic signaling pathway in response to oxidative stress; NROSA: negative regulation of oxidative stress-induced intrinsic apoptotic signaling pathway; PROSA: positive regulation of oxidative stress-induced intrinsic apoptotic signaling pathway; PROSNA: positive regulation of oxidative stress-induced neuron intrinsic apoptotic signaling pathway; SDA: superoxide dismutase activity; ROSND: regulation of oxidative stress-induced neuron death; NROSND: negative regulation of oxidative stress-induced neuron death; RS: response to superoxide; NROSCD: negative regulation of oxidative stress-induced cell death; NRHPNA: negative regulation of hydrogen peroxide-induced neuron intrinsic apoptotic signaling pathway; NRAHP: negative regulation of intrinsic apoptotic signaling pathway in response to hydrogen peroxide; NRHPCD: negative regulation of hydrogen peroxide-induced cell death; PH: response to hydroperoxide; CPH: cellular response to hydroperoxide; SRLP: response to lipid hydroperoxide; PRTROS: positive regulation of transcription from RNA polymerase II promoter in response to oxidative stress; RRSR: regulation of removal of superoxide radicals.

^*^ P<0.05. ^**^ P<0.01.


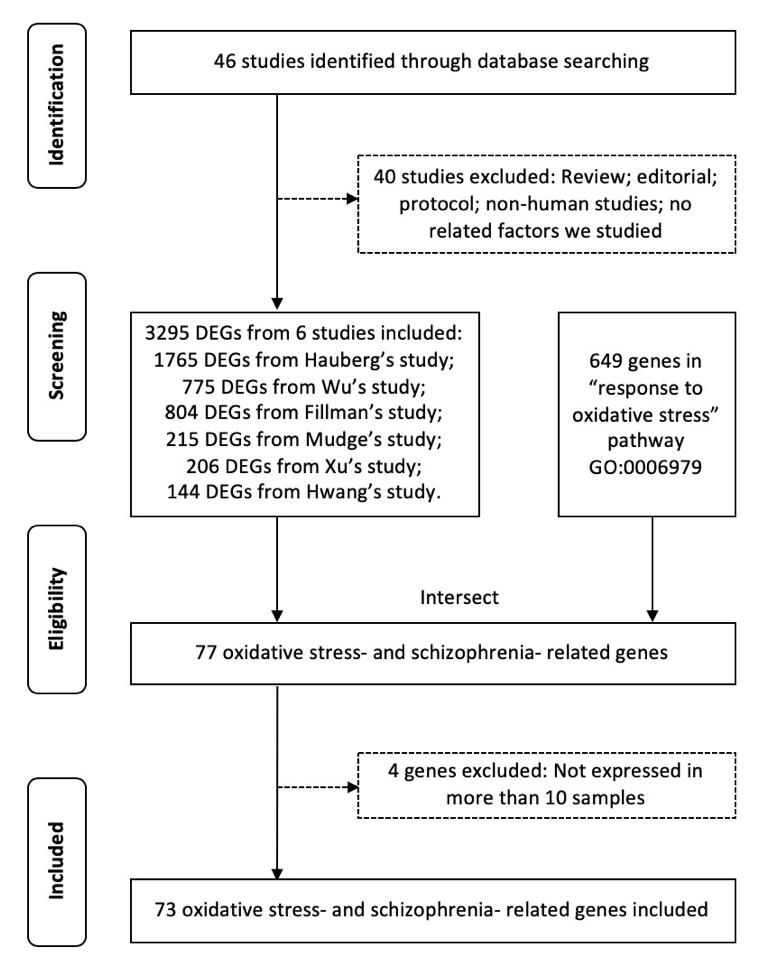


Figure S1. Flow diagram of the oxidative stress- and schizophrenia- related genes selection.
